# Finding crystal structures from few diffraction data by a combination of a random search with genetic algorithms

**DOI:** 10.1107/S0021889808020074

**Published:** 2008-07-16

**Authors:** Attilio Immirzi, Loredana Erra, Consiglia Tedesco

**Affiliations:** a Dipartimento di Chimica, Università di Salerno, I-84084 Fisciano (SA), Italy

**Keywords:** structural analysis, sparse diffraction data, random search algorithms, genetic algorithms, computer programs, *TRY*

## Abstract

A new procedure for performing structural analysis of crystalline materials from diffraction data, using internal coordinates, is described. For starting information only unit-cell content, space group, chemical formula, molecular connectivity and a limited amount of diffraction data are required. After first selecting a number of solutions using a Monte Carlo approach with severe filters, which reject the most unrealistic solutions, genetic algorithms (crossover and mutations) are applied. In fact, the initial selection step alone is, frequently, a powerful tool for discovering structures, without recourse to the genetic algorithms. The procedure, while suffering from the limitation that connectivity must be known, is effective in cases where direct methods are not applicable because the diffraction data are scarce, are limited to low diffraction angles or are missing in specific portions of the reciprocal space. The main features of the algorithm are described and examples of validation given. The routines are now available as part of the freely distributed general-purpose program *TRY*. The program is available on the Web at http://www.theochem.unisa.it/try.html.

## Abstract 2

A new procedure for performing structural analysis of crystalline materials from diffraction data by random search and genetic algorithms is described.

## 1. Introduction

A new computer program (*TRY*) for performing structure analysis and refinement using internal coordinates (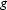) has been recently implemented (Immirzi, 2007*a* ▶,*b* ▶). There are numerous options for setting up a coarse structural model and then refining it by using the least-squares method.

*TRY* was designed for the study of difficult cases, where direct methods are unlikely to succeed because (i) there are many atoms with few and/or sparse data, (ii) the resolution is modest (*e.g.* in the case of powder diffraction) or (iii) there is a systematic lack of measurements in some regions of the reciprocal space [as in high-pressure studies with diamond anvil cells; see recent reviews by Katrusiak (2008 ▶) and Grochala *et al.* (2007 ▶)]. These drawbacks are also present in polymer crystallography.

To deal with such cases, we have introduced a new structure determination option in *TRY*. The procedure is applicable when the crystal symmetry and unit-cell content are known (likewise with direct methods), and, in addition, the atom connectivity is known. Uncertainty in the conformation, on the other hand, is not a problem.

The procedure consists of a wide-range ‘random walking’ in the internal coordinate space (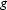 space), hunting for ‘reasonable solutions’, followed by ‘breeding’ among the solutions found using genetic mechanisms (crossover and mutations). In addition, the procedure has been strengthened by adding a routine for ‘improving’ the hunted solutions. In fact, the procedure is so robust that frequently the true structures can be found without recourse to the genetic algorithms.

When the procedure was applied to four known molecular structures, using only measured structure factors at low diffraction angles, the correct solution was found in each case; in two cases it was found directly from the initial set of random trials.

The procedure can be used as a preliminary step not just for genetic algorithms (Kariuki *et al.*, 1997 ▶; Harris *et al.*, 1998 ▶; Shankland *et al.*, 1998 ▶; Cheung & Harris, 2006 ▶) but also with other global optimization algorithms, such as simulated annealing (David *et al.*, 1998 ▶, 2003 ▶; Coelho, 2000 ▶; Pagola *et al.*, 2000 ▶) and parallel tempering (Favre-Nicolin & Černý, 2002 ▶).

The new molecular building algorithm, based on non­redundant internal coordinates, employs a strictly analytical procedure in all cases (Immirzi, 2007*a* ▶). This plays an important role in the 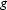-space random-walk procedure because all the internal coordinates are independent of each other and any valid random combination of the 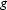 parameters produces a unique and well defined structure.

The candidate test cases considered were all single-crystal studies, but the number of input diffraction data was deliberately reduced to simulate instances where only a limited amount of reflection data is available.

We believe that the procedure has general applicability when there is a low data-to-unknown ratio and/or the data set is incomplete (high-pressure single-crystal data, fibre or powder data). While broadening the procedure to the Rietveld method has not yet been tested, it is entirely feasible.

It is important to emphasize that the procedure is applicable also when the crystal asymmteric unit is not an entire molecule but a fraction of it in the presence of molecular symmetry elements, and when the asymmetric unit consists of several molecules. The only problem is to specify correctly the connectivity (see below).

## 2. Main features of the new algorithm

Since the internal coordinates 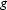 are continuous variables, computationally they must be treated using ‘real’ numbers. The dimensionality of these quantities may differ considerably (many are angles, some are lengths, some adimensional quantities) and their sensitivity may also be very different. At a crude level of structure analysis changes of angles of 1–2° should be of little significance; for translations the limit could be 0.1–0.2 Å. In addition, the various 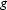 parameters span different intervals: bond lengths are substantially known *a priori* (customarily they are kept fixed); bond angles span very restricted intervals and can also be kept fixed in the structure-recognizing phase; rigid rotation angles and rigid translations for molecules span instead wide intervals. Molecular torsion angles span wide intervals in some cases (*e.g.* side-group rotations), while in others still they span rather restricted intervals (*e.g.* the conformational angles in closed rings).

For these reasons we have introduced a mechanism for varying 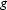 by small but finite steps. Trial structures are encoded as a bit-string assigning an appropriate number of bits to each 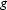, *i.e.* few bits for restricted-interval 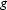 and more for wide-interval 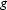. Angles in the range 0–360° can be encoded satisfactorily in 7–8 bits (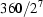 = 2.8°, 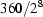 = 1.4° are reasonable steps). Fewer bits are required in encoding restricted-range torsion angles, and even fewer for encoding bond angles. The *cis*–*trans* isomerism for double bonds, if unknown, can be treated using a two-value torsion angle (0/180°), *i.e.* 1 bit only; if unknown, the chirality can also be encoded using 1 bit. Conformational angles in ethane-like situations (torsion angles restricted to −60, 60, 180°) can be treated using 2 bits.

The binary-encoded trials are integers much larger than 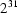, like the ordinary 4 byte integers used in all commercial computers, and also larger than 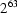 if 8 byte integers are allowed (as certain compilers do). A 512 bit size (64 bytes) has been assumed. If, for example, there are five 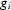 values and the number of bits dedicated to each one is 7, 6, 3, 4 and 6, the bit-string representing a trial structure (braces are used to group bits referring to a single 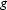 value) is
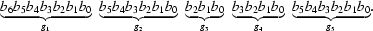
Each group of bits is an integer, which can be considered as a ‘digit’ in a rather unusual positional representation of numbers with variable base.

First of all, one must establish the number of bits (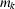) to assign to each variable 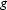 and the step size 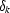. If 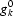 are the initial values for 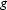 (the value of each 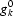 is arbitrary but it is the central point of the spanning interval), the possible values for 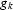 are 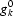, 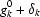, 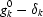, 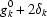, 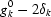, and so on (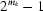 values). In our limited experience, using small values for 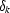 and relatively large ones for 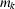 is convenient. In encoding a bond angle, for example, 3 bits and a 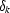 of 0.5° are sufficient for varying the angle in an 8° interval. By contrast, in encoding an unrestricted torsion angle, a larger 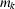 is required, *e.g.* 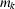 = 7 or 8 (see above). In encoding the overall rotation angles of a large molecule a finer resolution is appropriate since a small change of these angles may produce large effects. Of course this structure-encoding algorithm has been con­ceived both for performing the 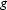-space random search and for carrying out the genetic combinations of selected structures.

Let us give a very simple example: methyl benzyl ether. At the structure elucidation level one can ignore H atoms and use as a model the nine-atom skeleton C—O—C—Ph (Ph is the phenyl ring). According to the known 3*N* − 6 rule, at a mol­ecular level there are 21 internal nonredundant coordinates, of which nine are bond lengths and 12 are bond and torsion angles. At a coarse level one assumes ‘canonical’ bond lengths, a regularly hexagonal aromatic ring and the coplanarity of the methylene C atom with the Ph ring, with a C—C
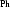
—C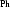 bond angle of 120°, thus there are only two bond angles (b.a.’s) and two torsion angles to be assigned. A good building plan, with a rather fine mesh in 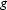 space, could be 
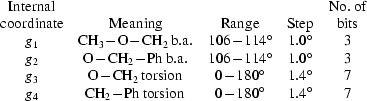

            *i.e.* a 20-bit encoding. Of course, the crystal structure requires six other 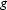 variables *i.e.* three molecular rotation angles and three translations, so that there are 10 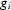 altogether. For the former a 7 bit encoding is sufficient; for the latter the number of bits must be chosen considering the unit-cell edges and a step of the order of 0.2 Å.

The procedure consists of three distinct stages: the first is simply a random walk in the 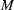-dimensional 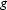 space (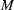 is the number of searched variables), the second can be described as a ‘local’ improvement process, and the third as a ‘breeding’ of structures, which mate with each other producing more or less reliable ‘child structures’. By repeating the breeding stage many times, the correct structure should emerge. In our limited experience, between four and eight breeding cycles seem sufficient. Each stage is performed by giving appropriate parameters regulating the child-structure selection.

### 2.1. Wide-range random walk: filters

The objective of the 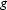-space random walk is to select around 100–200 more or less reliable trial structures attributing to the 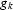 random values. The latter change by finite steps and the assigned values depend on the above-defined 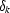 and 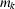. In detail, for each 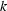 between 1 and 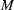, one generates a (real) random number 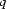 in the range 0–1, multiplies 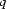 by 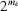, truncates to the nearest integer 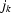 and assigns to 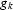 the appropriate value according to the above rule. Thus the initial 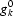 values are anything but critical inasmuch as 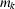 and 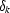 are high enough to span the 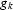 values in the appropriate interval. Indeed, millions of trials are necessary since not all combinations are ‘good’, only those surviving the appropriate ‘filters’.

The problem of filtering has recently been discussed by Hanson *et al.* (2007 ▶), who proposed the use of a parameter for assigning a ‘feasibility index’ to a random solution, based on the distances between nonbonded atoms compared with the sum of the van der Waals radii. The cited authors use this index not for rejecting *tout court* unfeasible trial structures but only for attributing a low probability parameter to them. We have preferred a different approach: to reject all the structures that fail to survive the filters. In this way (substantially consisting in rendering the Harris probability parameter a step function) the initial list of possible solutions is made up of reasonable structures only.

Of course, the severity of the filters is of crucial importance and experience is needed to establish practical rules. Appropriate conditions should reduce the number of selected trials to a fraction 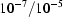 of the generated random numbers. We emphasize that the speed of the building plays an important role and that building using an analytical algorithm (as in *TRY*) is decidedly advantageous. Six operative filters have been implemented, as follows.

(1) The first filter rejects the trial whenever the selected combination of 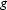 gives rise to some ‘building error’. Building errors can occur, for instance, when ring closure is attempted with incompatible torsion angles, or when a change of reference frame is performed on the basis of aligning three points. Another error condition occurs when one attempts to add an atom to a saturated C atom, imposing an 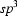 geometry with incompatible bond angles.

(2) The second filter is based on the molecular connectivity of the created structure, which is presumed to be known. The connectivity is defined by eight integers, or fewer in the simplest of cases: the number of atom pairs separated by one bond only, the number of atom pairs separated by two bonds *etc*., up to eight bonds. Naturally, other ways of defining connectivity could be devised. The trial is rejected if the random trial numbers do not match the correct connectivity codes. In fact, this filter is very fast and selective, particularly when the conformational freedom of the molecule is high.

(3) The third filter is based on the molecular conformation. The trial is rejected whenever an atom pair, separated by two or more bonds, is found with too short a separation (the limit is assigned by the user giving a value for two-bond-separated pairs and a value for pairs separated by more than two bonds). In practice, this filter removes strange shapes created by the random process, which should have high and improbable internal energy. In rigid-body problems, the searched internal coordinates are only molecular rotations and translations, making filters 1–3 unnecessary.

(4) The fourth filter is based on the number of chemical linkages between the asymmetric unit and the neighbouring atoms in the crystal; a ‘linkage’ is claimed whenever an atom-to-atom distance less than the sum of the covalent radii, plus a margin assigned by the user, is found. The number of linkages is expected to be zero in molecular substances in which the whole molecule is the asymmetric unit, greater than zero in symmetric molecules (the value depends on symmetry and on the occurrence of atoms in special positions) and two in linear polymers. Obviously in crosslinked structures the linkages can be more than two, but, at the moment, the program is not designed for these cases. Trials with an illegal number of chemical linkages are also rejected. This filter is also very selective, especially when the molecules are large.

(5) The fifth filter is based on the lattice energy, *E*, as evaluated from the packing distances and van der Waals radii. *TRY* adopts the Merck Molecular Force Field MMFF94 (see Halgren, 1992 ▶). For this filter a rather high value is suitable (*e.g.* 10–20 kcal mol^−1^; consider that the true values of lattice energy are negative). Caution is necessary in dealing with molecules with possible hydrogen bonds.

(6) The sixth filter is based on the 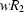 index {computed according to Sheldrick (2008 ▶), namely 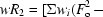 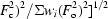}; trials with 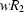 higher than an assigned value are rejected. Our initial experience suggests using unitary 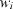 and setting a fairly high upper limit (*e.g.* 0.80–0.90). Even with slightly lower values (*e.g.* 0.75–0.80) the time taken to create the initial set of trial structures may be very prolonged. Of course the alternative use of the 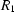 index (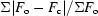) can be proposed, but it has not yet been thoroughly tested.

The ‘random’ search process can take a few hours or may need to run overnight. The duration could be significantly reduced by using parallel processing. The time taken depends on the number of selected trials and how the filtering parameters are assigned. In fact, filtering is particularly effective, even in complicated molecules. The number of reflections also plays a role, but a relatively modest one, since structure factors and 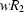 index are computed only for trials surviving filters 1–5 (see above). Finally, the selected trials are ordered by increasing 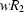 values.

### 2.2. Improving trials

The selected trials, which are of course very sparse points in 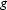 space, can be locally improved. This can be achieved using methods such as the ‘steepest descent’ or ‘conjugate gradients’ (Press *et al.*, 1992 ▶), or by simply looking at the nearest points in 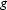 space case by case.

For the time being, this last procedure has been adopted. The program considers either the 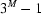 or the 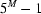 or the 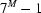 adjacent points and moves to the most favourable one on the grounds of the 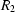 value. If 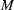 is large, 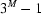 (and still more 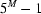 and 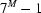) may become so large that it is impractical to look at all adjacent points. We have obtained good results using a Monte Carlo approach, selecting at random, say, 500–1000 neighbouring points. When this ‘local random walking’ is used, the above filters are again applied and play an especially important role if the chosen 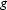-space mesh is coarse. Either way, this ‘improvement’ phase may be lengthy, but it is effective as the 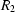 values may decrease considerably. At the end of this phase the structures are again ordered by increasing 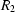 values. Rather frequently the trial improvement is so sharp that the correct solution emerges without recourse to the genetic algorithms.

### 2.3. Genetic algorithms

The genetic algorithms implemented in *TRY* are based on the consolidated breeding procedures known as ‘crossover’ and ‘mutation’. In addition, during the breeding phase a severe filtering strategy has been adopted using the same rules as discussed above. The upper limits for lattice energy and 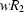 index may of course be distinct from the limits used in the search phase.

In a breeding cycle an assigned number (*e.g.* 30–50) of the best selected structures are mated with each other, selecting 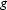 (either a single 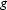 value chosen at random or all the 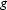 values in turn) and performing a ‘crossover’ (interchanging the selected 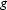 between the mating structures) and then queuing the resulting child structures in a list, provided filters are respected. In addition, ‘mutations’ can also be performed, and, in this case, not two but four child structures are produced by a given coupling. Mutations consist of selecting, at random, part of the binary encoded string and changing 1 to 0 or *vice versa*. Once mating is concluded, the whole list of structures is again sorted in ascending 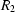 index order. We are also studying the use of alternative figures of merit, *e.g.* molecular energy, lattice energy and combinations thereof.

The breeding cycle is performed repeatedly, possibly using decreasing 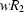 and energy limits. In our experience, after some four–six cycles the first say ∼40 solutions are almost indistinguishable and the true solution can be easily identified using a least-squares refinement. The choice of the filtering parameters (upper limits for 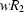 and lattice energy) is a critical point for which much experience must be accumulated. From our limited experience we would suggest giving a 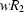 upper limit a little higher than the minimum; one observes typically that only a few child structures are selected in the first breeding cycle, while numerous child structures are selected in the subsequent cycles.

## 3. Program validation

The procedure has been tested by considering four known structures, all studied using single-crystal techniques and filed in the Cambridge Structural Database (Allen, 1998 ▶). Rather than use all the available diffraction data, a reduced data set was considered by excluding data at the higher diffraction angles. The data were deliberately reduced so that direct methods fail.

**Figure 1:**
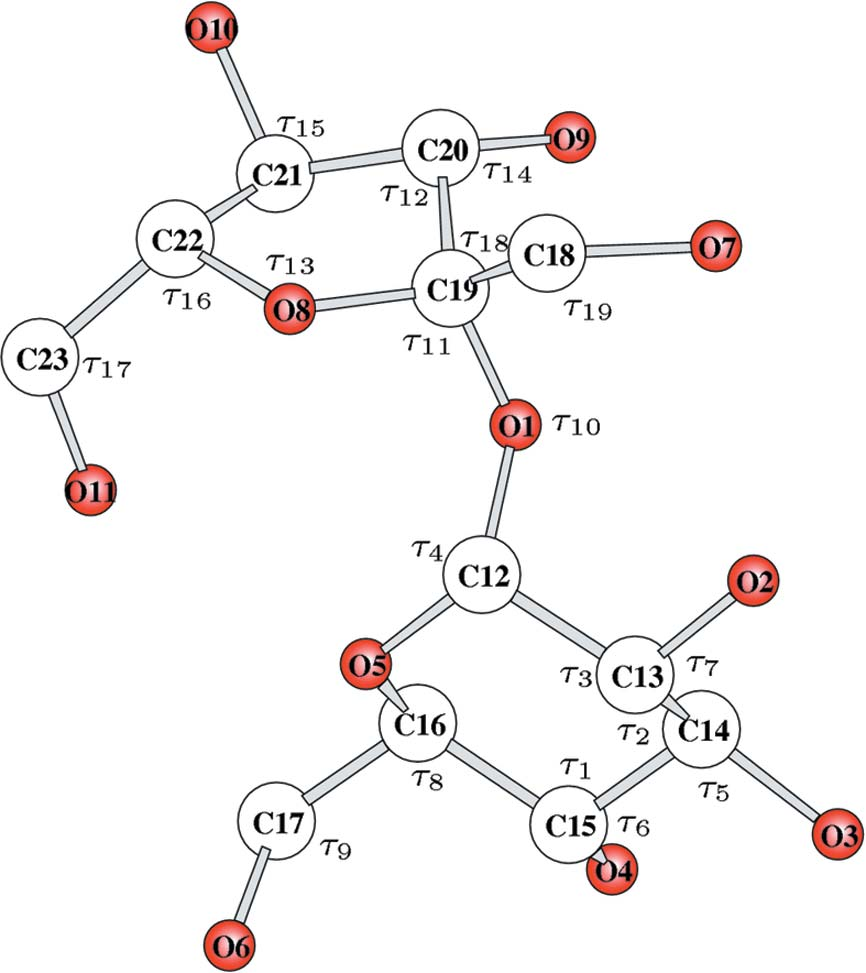
Molecular model for sucrose. Bond angles (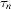) are shown. Torsion angles (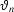) are listed in Table 1 ▶.

The building commands (see the supplementary materials^1^ [App appa]) show that molecular building is based on fixed bond lengths (defined as numerical constants), fixed bond angles 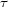 (defined symbolically), and variable torsion angles 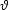 or bending angles 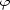 (also defined symbolically). The parameters 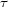 are defined in Figs. 1 ▶–4 ▶ ▶ ▶, and 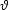 and 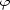 in Table 1 ▶ and in the supplementary materials (Tables S1–S4). H atoms are always neglected.

The working conditions and the bit-encoding mode (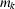 and 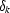 parameters and span intervals) are summarized in the same tables. Torsion angles 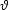 and out-of-plane bending angles 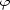 [used in dealing with closed rings; see Immirzi (2007*a* ▶)] were kept fixed in some cases. In other cases they are searched for, encoding them with an appropriate number of bits (see tables) distinguishing between angles internal to the rings, which span modest intervals; angles between rings, spanning a full 0–360° interval; angles controlling the position of side groups, spanning medium-sized intervals; and molecular rotation angles, spanning the widest intervals. For overall translations 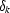 and 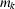 must be chosen by considering the lattice constants and the crystal symmetry.

In two out of the four cases, the genetic stage proved to be unnecessary as the correct solution (identified by comparison with the published one) was found from the first solutions selected. In all cases the least-squares method (refining of course the internal coordinates) gave a unique solution with an 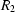 index close to the published one.

### 3.1. Sucrose, C_12_H_22_O_11_

Sucrose (Hynes & Page, 1991 ▶) has also been used for testing the special procedure implemented in *TRY* for modelling molecules with flexible rings (Immirzi, 2007*a* ▶). As discussed in the quoted article, sucrose can be modelled (at fixed bond lengths) using 44 internal coordinates, of which 24 are bond angles (excluded from the search), 18 torsion angles, two bending angles and five rototranslation parameters. Altogether 107 bits are used for the binary encoding of the structure. Random walking was performed by considering the 255 reflections (among the 1140 filed in the IUCr archives) with a 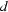 spacing higher than 1.7 Å.

The procedure has been applied by considering 25 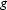 parameters in all. The result was that, creating the initial population (100 trials) with rather severe filters (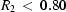, lattice energy 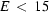
 kcal mol^−1^) and performing local improvement of the trials as discussed above (2000 points), the correct solution emerges without any recourse to genetic algorithms.

### 3.2. (+)-3,12-Dioxo-5β-cholanic acid, C_24_H_36_O_4_

A rather difficult candidate was selected for the second test. This substance (Kikolsky *et al.*, 2006 ▶) crystallizes as a mol­ecular compound of two conformers, which differ in the conformation of the side –COOH groups. In order to limit the number of internal coordinates the structure was analysed under the hypothesis that the central 19-atom unit (cyclopentaneperhydrophenantrene and the two attached methyl groups) has the same molecular structure for the two independent molecules and that this structure (common to all steroids) is known.

The building of this structure (at fixed bond lengths, and excluding the central 19-atom unit) requires eight bond angles (the same for the homologous terms), 4 + 4 torsion angles and 11 rototranslation parameters. Bond angles were not included in the search. To the 19 searched parameters the appropriate number of bits given in Table S2 were assigned (102 bits altogether). Random walking was performed by considering the 556 reflections (among the 5723 provided in the .fcf file) with 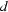 spacing higher than 1.5 Å. Once again, the correct structure was found by selecting 100 random trials and performing a local improvement.

### 3.3. c[-Pro-Thr-Aib-(S)β^3^-hPHe-Abu]

**Figure 3:**
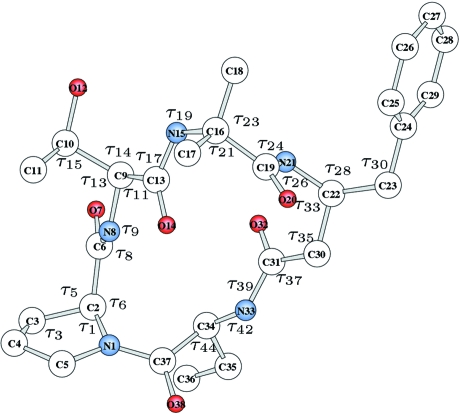
Molecular model for *c*[-Pro-Thr-Aib-(*S*)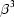-hPHe-Abu]. Bond angles (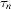) are shown. Torsion angles (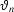) are listed in Table S3.

This synthetic cyclopeptide, related to the family of astins (Rossi *et al.*, 2004 ▶), with molecular formula 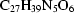
·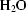, has been studied by X-ray diffraction [the uncoded 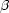-amino acid (*S*)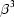-hPHe has the formula 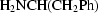 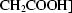. The present test is based on 711 unique reflections with 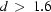
 Å belonging to the 2971 measured reflections. The molecule can be built (at fixed bond lengths) using 24 bond angles, defined in Fig. 3 ▶ (not included in the search), 20 torsion angles and two bending angles, defined in Table S3, where the number of bits, step size and range for each searched variable are also given. In addition the fractional coordinates of the solvent water molecule (O atom) are considered as independent variables. Note that wide intervals for the torsion angles were assumed, except for the peptide torsion for which a 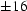
° range was considered, since these angles are systematically close to 180°. Altogether 163 bits were used for encoding the whole structure.

In this case the random search was not sufficient for finding the correct solution among 80 trials selected and locally improved; five or six breeding cycles were necessary for finding the structure.

### 3.4. 4′-Acetylbenzo-15-crown-5 2-naphthyloxyacetylhydrazone

**Figure 4:**
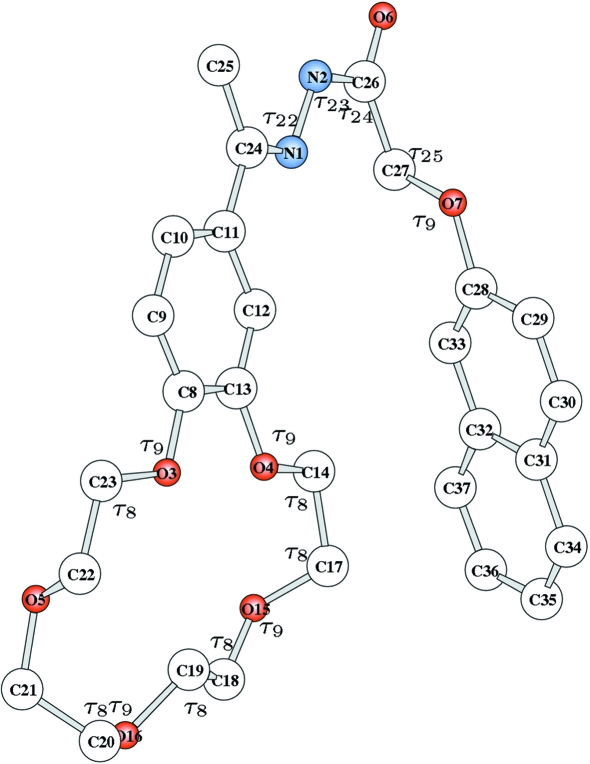
Molecular model for 4′-acetylbenzo-15-crown-5 2-naphthyloxyacetyl­hydrazone. Bond angles (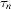) are shown. Torsion angles (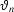) are listed in Table S4.

This rather complicated and conformationally very flexible molecule with formula 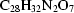 (Wei *et al.*, 2004 ▶) was considered for the last test. This was based on 807 unique reflections belonging to the 5262 measured reflections with 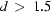
 Å. The molecule can be built (at fixed bond lengths) using five bond angles defined in Fig. 4 ▶ (not included in the search), 17 torsion angles and one bending angle defined in Table S4, where the number of bits, step size and range for each searched variable are also given. In all, 152 bits were used to encode the entire structure. In this case the structure was found by first selecting and improving 80 random structures, and then performing a systematic breeding among structures (crossover of all genes and mutations) with an acceptance level of 0.70 for 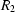 and 10 kcal for lattice energy. Five or six cycles of breeding were sufficient.

## 4. Conclusions

The test structures that have been described have all been selected from non-trivial cases and have given consistently encouraging results. The low number of data used suggests that powder diffraction problems should also be treatable. The ultimate validation of the new procedure will come, of course, by discovering some authentic new structures.

The procedure is actually programmed by considering the 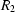 index as a ‘figure of merit’, while alternative figures of merit should be considered. A desirable next stage in the development of the procedure would be to make the program more ‘user friendly’. Presently, the user has to make rather a lot of decisions. However, before introducing such automation it would be worthwhile testing the program under a wider range of conditions. Naturally, the authors are open to suggestions for improvements. The program is available on the Web at http://www.theochem.unisa.it/try.html.

## Supplementary Material

Supplementary material file. DOI: 10.1107/S0021889808020074/kk5026sup1.pdf

## Figures and Tables

**Figure 2:**
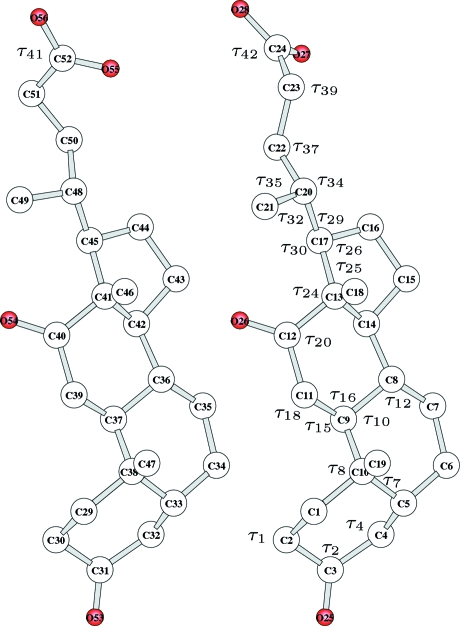
Molecular model for cholanic acid. Bond angles (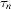) are shown. Torsion angles (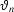) are listed in Table S2.

**Table 1:** Data for sucrose

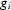	Definition		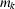	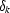	Span interval
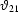	C16—C15—C14—C13		3	2.8°	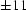 °
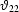	C15—C14—C13—C12		3	2.8°	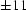 °
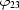	–		3	2.8°	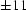 °
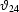	C16—O5—C12—O1		3	2.8°	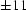 °
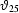	C16—C15—C14—O3		3	2.8°	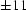 °
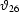	C13—C14—C15—O4		3	2.8°	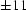 °
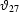	C15—C14—C13—O2		3	2.8°	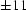 °
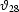	C14—C15—C16—C17		4	2.8°	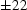 °
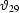	C15—C16—C17—O6		4	2.8°	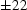 °
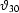	C13—C12—O1—C19		7	2.8°	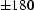 °
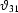	C12—O1—C19—C20		7	2.8°	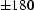 °
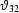	O1—C19—C20—C21		7	2.8°	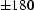 °
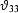	C19—C20—C21—C22		3	2.8°	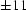 °
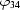	–		3	2.8°	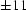 °
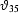	O8—C19—C20—O9		4	2.8°	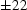 °
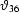	C19—C20—C21—O10		4	2.8°	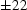 °
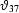	C19—O8—C22—C23		4	2.8°	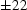 °
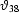	O8—C22—C23—O11		4	2.8°	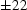 °
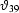	C21—C20—C19—C18		4	2.8°	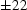 °
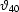	C20—C19—C18—O7		4	2.8°	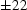 °
					
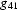 ( 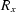 )			7	2.8°	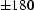 °
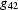 ( 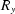 )			7	2.8°	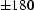 °
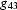 ( 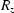 )			7	2.8°	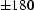 °
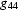 ( 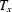 )			5	0.170	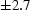 Å
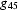 ( 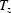 )			4	0.272	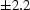 Å

## Appendix A Building symbolic language

The commands for building the molecular structures considered in this article are given. As supplementary material the complete files for doing structure resolution are supplied.

In all cases the bond-lengths are kept fixed and indicated as numerical constants; bond-angles , torsion-angles , and bending-angles  as symbols (), H atoms were always neglected. Info after exclamation mark ! is comment given for reader convenience. Commands obey to the syntax of the Building Symbolic Language () extensively described in the *TRY* manual. Note that the first 4-character string always denotes the kind of construction. Q-atoms are ‘pseudo-atoms’ used in the building process, but not included in the structure. Q-atoms may also be other entities like vectors, scalars, *etc*. since they are also used to store the results of calculations, as an example the command  refers the group of 23 atoms starting from C1 to its own inertial frame. The transformation matrix is loaded into Q50, Q51, Q52. The last digit is related to output options.

## Footnotes

1 Structure resolutions are available from the IUCr electronic archives (Reference: KK5026). Services for accessing these data are described at the back of the journal.
