# Supplementary material for: Finding crystal structures from few diffraction data by a combination of a random search with genetic algorithms
Source: J Appl Crystallogr. 2008 Jul 16;41(Pt 4):784–90. doi: 10.1107/S0021889808020074 (PMC2483486; doi:10.1107/S0021889808020074)
Supplement: Supplementary file 1 [file j-41-00784-sup1.pdf]

Supplementary Material for
Finding Crystal Structures From Few Diffraction Data
by a Combination of a Random Search with Genetic Algorithms

by Attilio Immirzi*, Loredana Erra, and Consiglia Tedesco

Dipartimento di Chimica, Università di Salerno
Fisciano (SA), I-84084, Italy

e-mail: aimmirzi@unisa.it

INDEX

1. The Building Symbolic Language for the presented structures 2

2. Tables with g-space search parameters and related values 6

1. The Building Symbolic Language for the presented structures.

The commands for building the molecular structures considered in the article are given. In all cases the bond-lengths are kept fixed and indicated as numerical constants; bond-angles τ, torsion-angles ϑ, and bending-angles φ as symbols (`gi`), H atoms were always neglected. Info after exclamation mark ! is comment given for reader convenience. Commands obey to the syntax of the Building Symbolic Language (`BSL`) extensively described in the `TRY` manual. Note that the first 4-character string always denotes the kind of construction. Q-atoms are "pseudo-atoms" used in the building process, but not included in the structure. Q-atoms may also be other entities like vectors, scalars, etc. since they are also used to store the results of calculations, as an example the command `iner K1 U2 n3 n4` refers the group of 23 atoms starting from C1 to its own inertial frame. The transformation matrix is loaded into Q50, Q51, Q52. The last digit is related to output options.

Sucrose

```
chbe C16  C15  C14        1.521   1.521   g1   !  begin from pyranose
setx C16  C15  C14  C13   1.521   g2      g21  !  2 z-matrix + 1 flap
setx C15  C14  C13  C12   1.521   g3      g22  !  are necessary
flap C15  C16  C12  O5    1.427   1.427   g23  !  close the ring
setx C16  O5   C12  O1    1.427   g4      g24  !  O1 is the bridge to furanose
setx C16  C15  C14  O3    1.427   g5      g25  !  set side O3
setx C13  C14  C15  O4    1.427   g6      g26  !  set side O4
setx C15  C14  C13  O2    1.427   g7      g27  !  set side O2
setx C14  C15  C16  C17   1.521   g8      g28  !  set side C17
setx C15  C16  C17  O6    1.427   g9      g29  !  set side O6
setx C13  C12  O1   C19   1.427   g10     g30  !  build furanose
setx C12  O1   C19  C20   1.521   g11     g31  !  3 z-matrix + 1 flap
setx O1   C19  C20  C21   1.521   g12     g32  !  are necessary
setx C19  C20  C21  C22   1.521   g13     g33  !
flap C21  C22  C19  O8    1.427   1.411   g34  !  close the ring
setx O8   C19  C20  O9    1.427   g14     g35  !  set side O9
setx C19  C20  C21  O10   1.427   g15     g36  !  set side O10
setx C19  O8   C22  C23   1.521   g16     g37  !  set side C23
setx O8   C22  C23  O11   1.427   g17     g38  !  set side O11
setx C21  C20  C19  C18   1.521   g18     g39  !  set side C18
setx C20  C19  C18  O7    1.427   g19     g40  !  set side O7
!  next commnd will orient the 2-atom molecule refering it to its own
!  inertial exes and placing the origin onto molecular centre.  This
!  operation, non strictly necessary, is useful since it reduces the
!  correlations among rotation angles in LS refinement.
iner C1   Q50  23   0    !  refer the molecule to its inertial frame
rtax 1    C1   23   g41  !  rotation of the whole molecule about x axis
rtax 2    C1   23   g42  !  rotation of the whole molecule about y axis
rtax 3    C1   23   g43  !  rotation of the whole molecule about z axis
ldat Q60  g44  0    g45  !  x,z=coordn.  of the centre; z (=0) is origin fixing
shft Q60  C1   23        !  shifts the molecule
end                      !  end building
```

(+)-3,12-Dioxo-5β-cholanic acid

```
!  building the 19-atom moiety common to the two molecules of the asymmetric unit
!  Ring I
chbe  C1   C2  C3   1.54 1.54   g1       !  begin from ring I (6 atoms)
setx  C1   C2  C3   C4   1.54   g2   g3  !  set C4 by z-matrix
setx  C2   C3  C4   C5   1.54   g4   g5  !  set C5 by z-matrix
flap  C2   C1  C5   C10  1.54   1.54 g6  !  set C10 by flap procedure
!  Ring II condensed
tets  C5   C10 C1   C9   1.54   g7   g8   g9 !  set C9 according to tetrahedral geometry
setx  C5   C10 C9   C8   1.54   g10  g11     !  set C8 by z-matrix
setx  C10  C9  C8   C7   1.54   g12  g13     !  set C7 by z-matrix
flap  C8   C7  C5   C6   1.54   1.54 g14     !  set C6 by flap procedure
!  Ring III condensed
tets  C10  C9  C8   C11  1.54   g15  g16 g17 !  set C11 according to tetrahedral geometry
setx  C8   C9  C11  C12  1.54   g18  g19     !  set C12 by z-matrix
setx  C9   C11 C12  C13  1.54   g20  g21     !  set C13 by z-matrix
flap  C12  C13 C8   C14  1.54   1.54  g22    !  set C14 by flap procedure
!  Ring IV consensed
tets  C12  C13 C14  C17  1.54   g23  g24 g25 !  set C17 according to tetrahedral geometry
setx  C12  C13 C17  C16  1.54   g26  g27     !  set C16 by z-matrix
flap  C13  C14 C16  C15  1.54   1.54  g28    !  set C15 by flap procedure
tert  C1   C5   C9   C10  C19  g59  !  setup C19 completing C10 valence
tert  C12  C14  C17  C13  C18  g59  !  setup C18 completing C13 valence
iner  C1   Q90  19 0 !  transpose the 19-atom model into inertial frame
!  Copy the 19-atom moiety into the second set to create the second molecule
copy  C1   C29  18
!  complete A molecule
tets  C16  C17  C13  C20  1.54 g29  g30  g31 !  set C20 according to tetrahedral geometry
setx  C16  C17  C20  C21  1.54 g32  g33      !  set C21 by z-matrix
tets  C17  C20  C21  C22  1.54 g34  g35  g36 !  set C22 according to tetrahedral geometry
setx  C21  C20  C22  C23  1.54 g37  g38      !  set C23 by z-matrix
setx  C20  C22  C23  C24  1.54 g39  g40      !  set C24 by z-matrix
setx  C22  C23  C24  O28  1.31 g41  g42      !  set O28 by z-matrix
trig  C23  C24  O28  O27  1.22 0             !  set O27 according to C2v symmetry
trig  C2   C3   C4   O25  1.22 0             !  set O25 according to C2v symmetry
trig  C11  C12  C13  O26  1.20 0             !  set O26 according to C2v symmetry
!  roto-translation operation
rtax 1 C1   28  g45 !  rotation of A molecule about x
rtax 2 C1   28  g46 !  rotation of A molecule about y
rtax 3 C1   28  g47 !  rotation of A molecule about z
ldat Q84   g48  g49  g50 !  load in Q84 Tx, Ty, Tz
shft Q84   C1 28         !  translation of the molecule A
!  complete B molecule
tets  C44  C45  C41  C48  1.54 g29  g30  g31 !  set C48 according to tetrahedral geometry
setx  C44  C45  C48  C49  1.54 g32  g57      !  set C49 by z-matrix
tets  C45  C48  C49  C50  1.54 g34  g35  g36 !  set C50 according to tetrahedral geometry
setx  C49  C48  C50  C51  1.54 g37  g58      !  set C51 by z-matrix
setx  C48  C50  C51  C52  1.54 g39  g59      !  set C52 by z-matrix
setx  C50  C51  C52  O56  1.32 g41  g60      !  set O56 by z-matrix
trig  C51  C52  O56  O55  1.22 0             !  set O55 according to C2v symmetry
trig  C30  C31  C32  O53  1.22 0             !  set O53 according to C2v symmetry
trig  C39  C40  C41  O54  1.22 0             !  set O54 according to C2v symmetry
rtax 1 C29  28  g51 !  rotation of B molecule about x
rtax 2 C29  28  g52 !  rotation of B molecule about y
rtax 3 C29  28  g53 !  rotation of B molecule about z
ldat Q134  g54  g55  g56 !  load in Q134 Tx, Ty, Tz
shft Q134  C29  28 !  translation of the molecule B
end                !  end building
```

c[-Pro-Thr-Aib-(S)β³-hPHe-Abu] cyclopeptide

```
chbe  N1  C2  C3  1.491  1.505  g1           !  begin from proline ring
setx  N1  C2  C3  C4  1.452  g2  g3          !  set C4 by z-matrix
flap  C2  N1  C4  C5  1.455  1.490  g4       !  set C5 by flap procedure
tetr  N1  C2  C3  C6  1.521  g5  g6  -1      !  attach C6 tetrahedrally
setx  N1  C2  C6  N8  1.322  g7  g8          !  set N8 by Z-matrix
trig  C2  C6  N8  O7  1.241  0               !  set O7 according to C2v symmetry
setx  C2  C6  N8  C9  1.452  g9  g10         !  set C9 by z-matrix
setx  C6  N8  C9  C13 1.501  g11 g12         !  set C13 by z-matrix
tetr  N8  C9  C13 C10 1.555  g13 g14 1       !  attach C10 tetrahedrally
setx  C13 C9  C10 C11 1.515  g15 g16         !  set C11 by z-matrix
tetr  C9  C10 C11 O12 1.389  1.085 1.131 -1  !  attach O12 tetrahedrally
setx  N8  C9  C13 N15 1.314  g17 g18         !  set N15 by z-matrix
trig  C9  C13 N15 O14 1.241  0               !  set O14 according to C2v symmetry
setx  C9  C13 N15 C16 1.484  g19 g20         !  set C16 by z-matrix
setx  C13 N15 C16 C19 1.530  g21 g22         !  set C19 by z-matrix
sc2v  N15 C16 C19 C17 1.535  g23             !  set C17 and C18 with local C2v symmetry
setx  N15 C16 C19 N21 1.353  g24 g25         !  set N21 by z-matrix
trig  C16 C19 N21 O20 1.244  0               !  set O20 according to C2v geometry
setx  C16 C19 N21 C22 1.460  g26 g27         !  set C22 by z-matrix
setx  C19 N21 C22 C23 1.577  g28 g29         !  set C23 by z-matrix
setx  N21 C22 C23 C24 1.492  g30 g31         !  set C24 by z-matrix
phen  C23 C24 C22 1.400 g32                  !  build-up a phenyl with D6d simmetry
setx  C19 N21 C22 C30 1.501  g33 g34         !  set C30 by z-matrix
setx  N21 C22 C30 C31 1.495  g35 g36         !  set C31 by z-matrix
setx  C22 C30 C31 N33 1.323  g37 g38         !  set N33 by z-matrix
trig  C30 C31 N33 O32 1.256  0               !  set O32 according to C2v symmetry
setx  C30 C31 N33 C34 1.438  g39 g40         !  set C34 by z-matrix
flap  N33 C34 N1  C37 1.512  1.341  g41      !  set C37 by flap procedure
setx  C31 N33 C34 C35 1.523  g42 g43         !  set C35 by z-matrix
setx  N33 C34 C35 C36 1.516  g44 g45         !  set C36 by z-matrix
trig  C34 C37 N1  O38 1.255  0               !  set N1 according to C2v symmetry
iner  N1  Q210  38  0  !  transpose the molecule into inertial frame
rtax  1   N1  38  g47  !  rotation about x
rtax  2   N1  38  g48  !  rotation about y
rtax  3   N1  38  g49  !  rotation about z
ldat  Q90 g50 g51 g52  !  load in Q90 Tx, Ty, Tz
shft  Q90 N1  38       !  traslation of the molecule
end                    !  end building
```

4'-Acetylbenzo-15-crown-5 2-naphthyloxyacetylhydrazone

```
chbe C22 C23 O3      1.48  1.40 g8  !  begin from the crown ether ring
setx C22 C23 O3  C8  1.40  g9   g30 !  set C8 by z-matrix
phen O3  C8  C23     1.40       g31 !  build-up a phenyl with D6d simmetry
setx O3  C8  C13 O4  1.40 1.2   0.0 !  set O4 by z-matrix
setx C8  C13 O4  C14 1.40 g9    g33 !  set C14 by z-matrix
setx C13 O4  C14 C17 1.38 g8    g34 !  set C17 by z-matrix
setx O4  C14 C17 O15 1.40 g8    g35 !  set O15 by z-matrix
setx C14 C17 O15 C18 1.40 g9    g36 !  set C18 by z-matrix
setx C17 O15 C18 C19 1.48 g8    g37 !  set C19 by z-matrix
setx O15 C18 C19 O16 g2   g8    g38 !  set O16 by z-matrix
setx C18 C19 O16 C20 g2   g9    g39 !  set C20 by z-matrix
setx C19 O16 C20 C21 g1   g8    g40 !  set C21 by z-matrix
flap C20 C21 C22 O5  g2   g2    g41 !  set C5 by flap procedure
line C8  C11 C24 g1                 !  set C24 aligned to C11 and C8
setx C10 C11 C24 N1  g4   1.2   g42 !  set N1 by z-matrix
trig C11 C24 N1  C25 g1  0          !  set C25 according to C2v symmetry
setx C11 C24 N1  N2  g5  g22  g43   !  set N2 by z-matrix
```

```
setx C24 N1  N2  C26 g6  g23  g44   !  set C26 by z-matrix
setx N1  N2  C26 C27 g1  g24  g45   !  set C27 by z-matrix
trig N2  C26 C27 O6  g7  0          !  set O6 according to C2v symmetry
setx N2  C26 C27 O7  g2  g25  g46   !  set O7 by z-matrix
setx C26 C27 O7  C28 g2  g9   g47   !  set C28 by z-matrix
phen O7  C28 C27     1.40 g48       !  build-up a phenyl with D6d simmetry
trig C30 C31 C32 C34 1.40 0         !  set C32 according to C2v symmetry
trig C31 C32 C33 C37 1.40 0         !  set C37 according to C2v symmetry
setx C30 C31 C34 C35 1.40 1.2  1.8  !  set C35 by z-matrix
flap C34 C35 C37 C36 1.40 1.40 1.8  !  set C36 by flap procedure
iner N1  Q99 37  0  !  transpose the molecule into inertial frame
rtax 1  N1  37  g49 !  rotation about x
rtax 2  N1  37  g50 !  rotation about y
rtax 3  N1  37  g51 !  rotation about z
ldat Q97 g52  g53  g54 !  load in Q97 Tx, Ty, Tz
shft Q97 N1 37         !  translation of the molecule
end                    !  end building
```

2. Tables with g-space search parameters and related values.

Table S1. Data for sucrose

g_i	definition	m_k	δ_k	span interval
ϑ_{21}	C16-C15-C14-C13	3	2.8°	±11°
ϑ_{22}	C15-C14-C13-C12	3	2.8°	±11°
φ_{23}		3	2.8°	±11°
ϑ_{24}	C16-O5-C12-O1	3	2.8°	±11°
ϑ_{25}	C16-C15-C14-O3	3	2.8°	±11°
ϑ_{26}	C13-C14-C15-O4	3	2.8°	±11°
ϑ_{27}	C15-C14-C13-O2	3	2.8°	±11°
ϑ_{28}	C14-C15-C16-C17	4	2.8°	±22°
ϑ_{29}	C15-C16-C17-O6	4	2.8°	±22°
ϑ_{30}	C13-C12-O1-C19	7	2.8°	±180°
ϑ_{31}	C12-O1-C19-C20	7	2.8°	±180°
ϑ_{32}	O1-C19-C20-C21	7	2.8°	±180°
ϑ_{33}	C19-C20-C21-C22	3	2.8°	±11°
φ_{34}		3	2.8°	±11°
ϑ_{35}	O8-C19-C20-O9	4	2.8°	±22°
ϑ_{36}	C19-C20-C21-O10	4	2.8°	±22°
ϑ_{37}	C19-O8-C22-C23	4	2.8°	±22°
ϑ_{38}	O8-C22-C23-O11	4	2.8°	±22°
ϑ_{39}	C21-C20-C19-C18	4	2.8°	±22°
ϑ_{40}	C20-C19-C18-O7	4	2.8°	±22°
g_{41} (R_x)		7	2.8°	±180°
g_{42} (R_y)		7	2.8°	±180°
g_{43} (R_z)		7	2.8°	±180°
g_{44} (T_x)		5	0.170	±2.7 Å
g_{45} (T_z)		4	0.272	±2.2 Å

Table S2. Data for cholanic acid

g_i	definition	m_k	δ_k	span interval
ϑ_{33}	C16-C17-C20-C21	5	2.8°	±45°
ϑ_{38}	C21-C20-C22-C23	5	2.8°	±45°
ϑ_{40}	C20-C22-C23-C24	5	2.8°	±45°
ϑ_{42}	C22-C23-C24-O28	5	2.8°	±45°
ϑ_{57}	C44-C45-C48-C49	5	2.8°	±45°
ϑ_{58}	C49-C48-C50-C51	5	2.8°	±45°
ϑ_{59}	C48-C50-C51-C52	5	2.8°	±45°
ϑ_{60}	C50-C51-C52-C56	5	2.8°	±45°
g_{45} (R_{xA})		6	1.4°	±45°
g_{46} (R_{yA})		6	1.4°	±45°
g_{47} (R_{zA})		6	1.4°	±45°
g_{48} (T_{xA})		5	0.20 Å	±3.2Å
g_{50} (T_{zA})		6	0.18 Å	±5.8Å
g_{51} (R_{xB})		6	1.4°	±45°
g_{52} (R_{yB})		6	1.4°	±45°
g_{53} (R_{zB})		6	1.4°	±45°
g_{54} (T_{xB})		5	0.20 Å	±3.2Å
g_{55} (T_{yB})		4	0.22 Å	±1.8Å
g_{56} (T_{zB})		6	0.18 Å	±5.8Å

Table S3. Data for c[-Pro-Thr-Aib-(S)β^3-hPHe-Abu]

g_i	definition	m_k	δ_k	span interval
ϑ_3	N1-C2-C3-C4	3	2.0°	±8°
φ_4	C2-N1-C4-C5	5	2.0°	±32°
ϑ_8	N1-C2-C6-N8	6	2.0°	±64°
ϑ_{10}	C2-C6-N8-O7	4	2.0°	±16°
ϑ_{12}	C6-N8-C9-C13	6	2.0°	±64°
ϑ_{16}	C13-C9-C10-C11	6	2.0°	±64°
ϑ_{18}	N8-C9-C13-N15	6	2.0°	±64°
ϑ_{20}	C9-C13-N15-C16	6	2.0°	±64°
ϑ_{22}	C13-N15-C16-C19	6	2.0°	±64°
ϑ_{25}	N15-C16-C19-N21	6	2.0°	±64°
ϑ_{27}	C16-C19-N21-C22	4	2.0°	±16°
ϑ_{29}	C19-N21-C22-C23	6	2.0°	±64°
ϑ_{31}	N21-C22-C23-C24	6	2.0°	±64°
ϑ_{32}	C22-C23-C24-C25	6	1.4°	±90°
ϑ_{34}	C19-C21-C22-C30	6	2.0°	±64°
ϑ_{36}	N21-C22-C23-C24	6	2.0°	±64°
ϑ_{38}	C22-C30-C31-N33	6	2.0°	±64°
ϑ_{40}	C30-C31-N33-C34	4	2.0°	±16°
ϑ_{41}	N33-C34-N1-C37	7	2.0°	±128°
ϑ_{43}	C31-N33-C34-C35	6	2.0°	±64°
ϑ_{45}	N33-C34-C35-C36	6	2.0°	±64°
g_{47} (R_x)		7	1.4°	±90°
g_{48} (R_y)		7	1.4°	±90°
g_{49} (R_z)		7	1.4°	±90°
g_{50} (T_x)		5	0.15Å	±2.4Å
g_{51} (T_y)		5	0.15Å	±2.4Å
g_{53} (x_w)		5	0.10Å	±0.16Å
g_{54} (y_w)		5	0.10Å	±0.16Å
g_{55} (z_w)		5	0.10Å	±0.16Å

Table S4. Data for 4'-Acetylbenzo-15-crown-5 2-naphthyloxyacetylhydrazone

g_i	definition	m_k	δ_k	span interval
ϑ_{30}	C22-C23-O3-C8	7	2.0°	±128°
ϑ_{31}	phenyl	7	2.0°	±128°
ϑ_{33}	C8-C13-O4-C14	7	2.0°	±128°
ϑ_{34}	C13-O4-C14-C17	7	2.0°	±128°
ϑ_{35}	O4-C14-C17-O15	7	2.8°	±128°
ϑ_{36}	C14-C17-O15-C18	7	2.8°	±128°
ϑ_{37}	C17-O15-C18-C19	7	2.8°	±128°
ϑ_{38}	O15-C18-C19-O16	7	2.8°	±128°
ϑ_{39}	C18-C19-O16-C20	7	2.8°	±128°
ϑ_{40}	C19-O16-C20-C21	7	2.8°	±128°
φ_{41}	C20-C21-C22-O5	4	1.0°	±8°
ϑ_{42}	C10 C11 C24 N1	5	2.0°	±32°
ϑ_{43}	C11-C24-N1-N2	7	2.0°	±128°
ϑ_{44}	C24-N1-N2-C26	7	2.0°	±128°
ϑ_{45}	N1-N2-C26-C27	5	2.0°	±32°
ϑ_{46}	N2-C26-C27-O7	5	2.0°	±32°
ϑ_{47}	C26-C27-O7-C28	5	2.0°	±32°
ϑ_{48}	phenyl	6	2.0°	±64°
g_{49} (R_x)		7	2.0°	±128°
g_{50} (R_y)		7	2.0°	±128°
g_{51} (R_z)		7	2.0°	±128°
g_{52} (T_x)		5	0.137Å	±2.2 Å
g_{53} (T_y)		6	0.250Å	±8.0 Å
g_{54} (T_z)		5	0.187Å	±3.0 Å
